# Development and characterization of microsatellite loci in the *Centricnemus leucogrammus* weevil

**DOI:** 10.1007/s11033-012-2021-1

**Published:** 2012-10-13

**Authors:** Łukasz Kajtoch, Krystyna Nadachowska-Brzyska, Wiesław Babik

**Affiliations:** 1 Department of Experimental Zoology, Institute of Systematics and Evolution of Animals, Polish Academy of Science, Sławkowska 17 St., 31-016 Kraków, Poland; 2 Institute of Environmental Sciences, Jagiellonian University, Gronostajowa 7, 30-387 Kraków, Poland; 3 Department of Evolutionary Biology, Evolutionary Biology Centre, Uppsala University, Norbyvägen 18D, 75236 Uppsala, Sweden

**Keywords:** Coleoptera, Curculionidae, Conservation, Population genetics, Short tandem repeats, 454 Sequencing

## Abstract

*Centricnemus leucogrammus* is a weevil characteristic of European xerothermic habitats and steppes. The species was probably more widespread during the Pleistocene glaciations, while its current distribution is limited to “warm-stage refugia.” It may be regarded as a typical representative of flightless xerothermophilous beetles. Previous studies concentrated on its genetic variation using mitochondrial genes. Here, we identified, tested and characterized 24 polymorphic microsatellite loci with the use of 454 sequencing of microsatellite enriched genomic libraries. The new set of loci will be used in studies on the population structure of this weevil and may provide valuable information for its conservation.

## Introduction

*Centricnemus leucogrammus* (Germar 1824) (Curculionidae: Entiminae: Peritelini) is a weevil characteristic of European xerothermic habitats and steppes. The diploid number of chromosomes in *C. leucogrammus* is 22 [1]. The species is apterous, thus its mobility is very limited. The distribution of *C. leucogrammus* is restricted to dry and warm xerothermic grasslands and steppes and is not limited by host plant availability because the species is polyphagous. The main range of this beetle extends from the Black Sea to the highlands of Ukraine and central Poland; however, its distribution is patchy. *C. leucogrammus* also occupies dry grasslands in the German uplands and in the Pannonian Basin, along the Danube valley. Isolated populations have been recently found in northern Poland in the lower Vistula and lower Oder valleys. The distribution in central Europe consists of a network of small populations, partially or fully isolated from each other. Despite its limited distribution, the species is quite abundant in suitable environments, which enables the collection of reasonably large samples for population studies. Evidence suggests that the present geographical range of this weevil has been strongly influenced by its history. Recent studies on the mitochondrial and nuclear diversity of *C. leucogrammus* populations from central and eastern Europe [2, 3] have revealed six mitochondrial and three nuclear lineages; however, populations from different regions often share similar haplotypes/genotypes. The pattern of mtDNA variation suggests that *C. leucogrammus* populations have probably existed in central and eastern Europe for at least 400,000 years. Apparently, the species has undergone multiple cycles of range contraction and expansion, which may have created an opportunity for mixing divergent genetic lineages. The species was probably more widespread during the Pleistocene glaciations, when climatic and environmental conditions favored dry grasslands [2, 4], while its current distribution is limited to “warm-stage refugia” [5].

The range of *C. leucogrammus* has been shrinking for the last few decades, especially on the western margin of its distribution (e.g., in Polish Silesia) but also in the main section of the range (Kajtoch Ł., unpublished data). This weevil may be regarded as a typical representative of flightless xerothermophilous beetles. Information about the genetic diversity of this species may be useful for the conservation and management of entire xerothermic communities, and in particular of invertebrate taxa of limited mobility. Xerothermic habitats are unique environments regarded as extrazonal remnants of glacial steppes; their communities are rich in rare and endemic species, especially insects (e.g., many flightless beetles). These habitats are threatened due to climatic and anthropogenic changes. Preliminary studies on phylogeography of steppic Coleoptera [3, 6, 7] suggest that they are structured geographically and populations of particular species are highly isolated from other regions but beetles within populations have limited or no genetic diversity. This may have important implications for conservation and management strategies for steppic species and entire species assemblages.

The ecological features and low mobility of *C. leucogrammus* make it an excellent model for studying the isolation of xerothermic patches on a local and regional scale. The availability of microsatellite loci for this taxon would facilitate such research. However most microsatellite loci reported so far for weevils have been developed for serious tree or cultivar pests only distantly related to *C. leucogrammus* (GenBank—http://www.ncbi.nlm.nih.gov/genbank/). As no microsatellites have so far been characterized for the genus *Centricnemus* or other Peritelini, it was necessary to develop a new set of loci to study patterns of isolation and gene flow in *C. leucogrammus*. Microsatellite loci are the markers of choice for such studies, clearly outperforming, due to high variation, simple inheritance and codominant nature, other genetic markers (such as RAPDs, AFLPs or mtDNA and nuclear DNA sequences).

## Materials and methods

A modified protocol of Glenn and Schable [8] in combination with 454 sequencing was used for the development of microsatellite loci. Genomic DNA (2.5 μg) from two individuals was digested with *Rsa*I enzyme (New England Biolabs) in a 20 μL reaction volume overnight at 37 °C. Thermal deactivation of the restriction enzyme was performed for 20 min at 80 °C. Double-stranded linkers (SuperSNX) were then ligated to DNA fragments in the presence of *Xmn*I restriction enzyme, which prevents linkers from dimerization. Linker-ligated DNA was used in a DynaBeads enrichment procedure. Two mixtures of 3′ biotinylated oligos were used in hybridization with DNA—Mix 1: (AG)_12_, (TG)_12_, (AAC)_6_, (AAG)_8_, (AAT)_12_, (ACT)_12_, (ATC)_8_ and Mix 2: (AAAT)_8_, (AACT)_8_, (AAGT)_8_, (ACAT)_8_, (AGAT)_8_. To capture DNA fragments with microsatellite sequences that were complementary to the microsatellite oligos, we used DynaBeads coated with streptavidin (Dynal, Oslo, Norway) and a magnetic particle concentrator (MPC, Dynal, Oslo, Norway). Six wash steps (two final steps were performed using solutions heated to 50 °C) were performed according to the manufacturer’s protocol. However, instead of precipitating the enriched fragments, a MinElute PCR purification kit (Qiagen) was used and the enriched DNA was eluted in 12 μL of AE buffer. After the enrichment, PCR was run with 2 μL of eluted DNA.

The following PCR protocol was used: 2.5 μL of 10× PCR buffer with (NH_4_)_2_SO_4_ (Fermentas), 2.0 μL of 25 mM MgCl2, 0.4 μL of 10 mM dNTP, 1.3 μL of SuperSNX-24F primer (100 mM), 0.2 μL of *Taq* (5 μ/μL) polymerase (Fermentas) and ddH_2_O to 25 μL. The cycling scheme was as follows: 94 °C for 2 min followed by 25 cycles of 95 °C for 20 s; 60 °C for 20 s, 72 °C for 90 s; the final extension was at 72 °C for 30 min. The PCR products were sequenced as a part of 454 Titanium run at the Functional Genomics Center Zurich.

We obtained 14,643 sequencing reads. Msatcommander [9] was used to search for sequencing reads containing at least eight dinucleotide or at least five perfect tri-, tetra- or penta-nucleotide repeat units, and 658 reads containing microsatellite repeats were found. This relatively low frequency of microsatellite repeats (4.5 % of reads) was probably caused by using only a single round of enrichment. We were able to design primers for 85 loci. Primer3 software (http://frodo.wi.mit.edu/primer3/; [10]), and in several cases also Primer-Blast software (http://www.ncbi.nlm.nih.gov/tools/primer-blast/; [10]), was used. To check for amplification and polymorphism of the selected loci, the method developed by Schuelke [11] was used. In this method, PCR is performed with three primers: a locus-specific forward primer with the M13 sequence tail at its 5′ end, a locus-specific reverse primer and a labeled M13 forward primer. The following PCR protocol was used for 10 μL reactions: 1 μL of genomic DNA (1–5 ng/μL), 3.25 μL of Qiagen multiplex PCR master mix (Qiagen, Hilden, Germany) and 0.2 μL of each primer (final concentration of each primer: 0.4 mM) and ddH_2_O to 10 mL. The cycling scheme was as follows: 94 °C for 15 followed by 40 cycles of 94 °C for 20 s, 55 °C for 90 s and 72 °C for 30 s; the final extension was at 72 °C for 10 min. PCR products were electrophoresed on an ABI 3130xl Genetic Analyser with GeneScan 500 LIZ size standard (Applied Biosystems). Allele sizes were determined using GeneMapper software (Applied Biosystems).

We examined 85 primer pairs. PCR products were obtained for 56 primer pairs, 16 of them could not be reliably scored and further 15 turned out to be monomorphic. Unambiguous scoring was possible for 24 polymorphic loci. Sequences of these microsatellite loci were deposited in GenBank (accession numbers JQ015190–JQ015214). These 24 loci were characterized in 60 individuals from populations of: western Ukraine (Podolian Upland), central Poland (Małopolska Upland), northern Poland (lower Vistula valley) and Slovakia-Moravia (Danube basin). We analyzed 16 individuals per population.

The number of alleles per locus per population, as well as the observed and expected heterozygosities, were calculated with Arlequin 3.1 [12]. Tests for departures from Hardy–Weinberg equilibrium (H–W), and tests for linkage disequilibria were performed using GENEPOP [13]. The type I error level was controlled through the false discovery rate (FDR) procedure [14], with FDR set to 0.05.

## Results and discussion

The number of alleles for polymorphic loci ranged from 2 to 18 and the observed heterozygosities from 0.0 to 0.875 (Table 1). Eight loci showed only limited polymorphism (2–3 alleles), 11 loci were moderately polymorphic (4–9 alleles) and three loci were highly polymorphic (11, 12 and 18 alleles for loci Cl-13643, Cl-14328 and Cl-14592, respectively). Some loci were monomorphic in particular regional populations: Ukrainian (Cl-270, Cl-38, Cl-1685, Cl-357), central Polish (Cl-542, Cl-1685, Cl-2144, Cl-10737), northern Polish (Cl-357, Cl-2144) and Slovakian-Moravian (Cl-2705, Cl-5756, Cl-1685) populations. Thus, these loci should be avoided while studying particular (local) populations.

**Table 1:** Characterization of polymorphic microsatellite loci in *C. leucogrammus*

Locus	454 Number	Primers	Repeat sequence	Pop.	Size range	*N*_A_	*H*_O_	*H*_E_
M30	Cl-2705	F: ATTTCGGGACACCCTGTAT	(ATC)6	U	146	1	0.0000	0.0000
				P	146–149	2	0.0625	0.1754
		R: CGTATTGGAGAGGCTTATGT						
				N	146–149	2	0.3750	0.3145
				S	146	1	0.0000	0.0000
				Total	146–149	2		
M3	Cl-226	F: TATGCACATCGAACAGGTCT	(AC)9	U	121–123	2	0.8750*	0.5081
		R: TTACAGCAAAACTCAATCAGG						
				P	119–123	3	0.6875	0.6270
				N	119–123	3	0.8750	0.6351
				S	119–123	3	0.750	0.5867
				Total	119–123	3		
M12	Cl-542	F: TCACATCGTATTGGTTCCTT	(AAAT)5	U	144–149	2	0.4375	0.4173
		R: ACGCAGAATGGGTAGTTCTT						
				P	144	1	0.0000	0.0000
				N	144–149	2	0.5625	0.5141
				S	144–148	2	0.1250	0.3145
				Total	144–149	3		
M8	Cl-38	F: AACCAGATCGAGCGACGACC	(GAT)6	U	116–122	3	0.1875	0.2843
		R: CCCTTCGCCCTTGCAGTTCT		P	121–127	2	0.1875	0.1754
				N	116–121	2	0.0625	0.1754
				S	121–127	2	0.0625	0.0625
				Total	116–127	4		
M11	Cl-19	F: TGACAAGATATTTCGAGCAA	(ACAT)5	U	168	1	0.0000	0.0000
		R: AAACTAACACGAGGGTAAACA						
				P	167–168	2	0.0000*	0.3871
				N	163–171	3	0.6875	0.5786
				S	163–171	3	0.3750	0.3306
				Total	163–171	4		
M44	Cl-2947	F: TTTTATCCGTCCATCATTAAA	(AAAT)5	U	111–126	4	0.1250*	0.7177
		R: CACTCTTCCCTACTCCCTTC						
				P	111–126	4	0.4375	0.5625
				N	111–125	4	0.3750*	0.6190
				S	111–125	3	0.5000	0.6129
				Total	111–126	6		
M1	Cl-171	F:CACTTTACATGCCGCTTATC	(GT)14	U	157–174	4	0.5000*	0.7601
		R: GCTTATGTATGAACGGAGGA						
				P	157–172	5	0.4375	0.3831
				N	165–174	3	0.4375	0.5060
				S	165–178	4	0.6875	0.5766
				Total	157–178	7		
M48	Cl-5756	F: ACTTCTCCACCTGGCAAG	(AAGT)5	U	128–131	2	0.0000*	0.2258
				P	126–131	3	0.1875*	0.5423
		R: TTTCATTACTCGACTTTACATTTC						
				N	128–131	2	0.1250	0.2258
				S	128	1	0.0000	0.000
				Total	126–131	3		
M27	Cl-1685	F: GGTTCAGTTCAGAGTGACAA	(GAT) 6	U	108	1	0.0000	0.0000
		R: TTCCGACGTTTTGTTCTTTA		P	108	1	0.0000	0.0000
				N	108–118	2	0.1250	0.1210
				S	108	1	0.0000	0.0000
				Total	108–118	2		
M4	Cl-357	F: TTCCTCCACAAGAGAATCG	(ATC)6	U	145	2	0.0000*	0.2258
		R: AGTAACTCTGCAAGGGAGGT						
				P	144–145	2	0.0000*	0.3871
				N	145	1	0.0000	0.0000
				S	138–145	2	0.4375	0.3528
				Total	138–145	3		
M34	Cl-2144	F: CCTCCACAAGAGAATCGGCG	(ATC)6	U	139–146	2	0.1250	0.1210
				P	146	1	0.0000	0.0000
		R: CTCTGCAAGGGAGGTTACACA		N	146	1	0.0000	0.0000
				S	139–146	2	0.3125	0.2722
				Total	139–146	2		
M35	Cl-2274	F: CCTAACAATAAAATAGAATTGCA	(AC)10	U	155–159	3	0.4375	0.6048
		R: TGGGAGGAGTAATCTGTGAC						
				P	155–159	3	0.3750	0.5867
				N	155–161	3	0.6250	0.6694
				S	155–162	4	0.6875	0.6915
				Total	155–162	5		
M45	Cl-2952	F: CACATCGTATTGGTTCCTTT	(AAAT)5	U	131–135	2	0.3750	0.3145
		R: TGGGTAGTTCTTTTGTACGC		P	120–131	2	0.1875	0.1754
				N	131–135	2	0.6250	0.4435
				S	120–135	3	0.3125	0.4536
				Total	120–135	3		
N11	Cl-15116	F: GAAAGGACACAATTTTTGC	(AGGT)17	U	240–256	7	0.4375*	0.7681
				P	240–256	6	0.5000	0.7399
		R: GCTGGAACAGGCATATTTT						
				N	228–244	4	0.2500*	0.5423
				S	228–252	6	0.4375*	0.6815
				Total	228–252	9		
N12	Cl-10737	F: CTCCGTAAGAAACACCAGTT	(GT)5ATGT	U	143–150	5	0.1875*	0.5020
		R: AAAGGACGAGTGAACACATT	GAT(GT)15					
				P	148	1	0.0000	0.0000
			GAG(GT)3	N	143–148	*2*	0.3125	0.2722
			AT(GT)4	S	143–148	2	0.0000*	0.4435
				Total	143–150	5		
N16	Cl-11923	F: AGTATTGTGACGCAGTGTGA	(CA)10CAT	U	163–167	3	0.4375	0.6028
		R: AGTGAAACTGTTCAGCGAAG	(CA)5	P	157–169	6	0.6875	0.6532
				N	165–167	2	0.3750	0.3871
				S	165–167	2	0.7500	0.4839
				Total	157–167	6		
N29	Cl-11170	F: CAGGTCTGGTAAGTGGGTTA	(GA)11	U	170–182	5	0.1875*	0.6290
		R: ACTGTGTGTGACGGGAAA		P	178–180	2	0.1250	0.1210
				N	174–182	4	0.1875*	0.5444
				S	167–182	6	0.5000	0.7198
				Total	167–182	8		
N36	Cl-14228	F: ACACCAGAAATAACGACAGC	(ATTT)10	U	141–151	4	0.2500	0.2359
		R: AAATATAGCTTAACGTGATACGG						
				P	137–151	3	0.3125*	0.5665
				N	141–151	4	0.3125*	0.7440
				S	137–151	4	0.1250*	0.6976
				Total	137–151	7		
N10	Cl-13643	F: AACGGGTTGATGTCTATCG	(GA)10	U	189–216	10	0.5625*	0.7843
		R: TGGTTTCCCTCTCTTCCTA		P	189–215	13	0.8125	0.8831
				N	200–206	3	0.1250*	0.5484
				S	189–208	8	0.5625	0.7540
				Total	189–216	18		
N13	Cl-14610	F: AACGTCACGAAGATCAAAGT	(ACTA)10	U	124–125	2	0.0000*	0.4435
		R: TGCTTTAATGACTTGGAGAAA		P	101–165	8	0.4375*	0.6673
				N	125–134	2	0.1875	0.1754
				S	125–134	2	0.3750	0.3145
				Total	101–134	8		
N18	Cl-14592	F: TTAATGTTAGGTTGGGCTGA	(AAAT)13	U	158–182	5	0.5000*	0.7480
		R: AAGTTACAGTTCAGAATAATTTGTG						
				P	145–174	4	0.3125	0.3327
				N	156–168	6	0.3125	0.8125
				S	158–174	6	0.3125	0.6573
				Total	145–182	12		
N35	Cl-14328	F: ATATGGGACTTCCTGTTTGT	(AAAT)8	U	94–112	10	0.5000*	0.8730
		R: TCGTGCTTAACTTACGTCCT						
				P	94–108	8	0.7500	0.7440
				N	98–106	5	0.0625*	0.6996
				S	98–101	7	0.1875*	0.8185
				Total	94–112	11		
N15	Cl-13806	F: ATTGAGGTTGTCGCTTTATG	(TATT)8	U	145–152	5	0.0000*	0.7264
		R: CGGCTAACCTATTACCGAGT		P	137–148	4	0.1667*	0.7065
				N	138–149	3	0.1667*	0.6212
				S	137–148	4	0.3333*	0.7391
				Total	137–152	7		
N34	Cl-11267	F: TTTGTAGAATGTATTAGGAGAACTAAA	(ATTC)14	U	150–160	2	0.2000*	0.2000
				P	144	2	0.0000*	0.4286
		R: AAATGAACTTGTATCTGTTATTGTG						
				N	144–150	1	0.0000*	0.0000
				Total	144–150	3		

*Pop.* populations, *U* western Ukraine, *P* central Poland, *N* northern Poland, *S* Slovakia-Moravia, *N*_A_ number of alleles observed, *H*_O_ observed heterozygosity, *H*_E_ expected heterozygosity

* Result significant at FDR = 0.05; indicates a significant departure from expected heterozygosity

Among 82 tests of H–W expectations, 37 were significant at FDR 0.05 (Table 1). Significant departures from H–W were detected in all studied populations only for two loci (Cl-13806 and Cl-11267). Also for these loci, amplification failed in multiple individuals suggesting a high frequency of null homozygotes. Thus, these two loci should be avoided in future studies.

For three loci significant departures from H–W expectations were detected in three populations, for seven loci in two populations and for three loci in a single population. The remaining eight loci did not show departures from H–W in any population (Table 1). The observed pattern of departures from H–W expectations may have been influenced by both the local presence of null alleles in moderate frequencies and by population processes (e.g., within some localities weevils were collected from several grasslands which may have been inhabited by distinct populations due to the extremely limited mobility of the species).

Out of 271 tests for linkage disequilibrium between pairs of loci, ten were significant at FDR 0.05. These significant linkage disequilibria were not, however, consistently observed for any pair of loci or any population, so they are probably not a consequence of physical linkage.

Taking into consideration above mentioned limitations the best set of microsatellite loci for studying *C. leucogrammus* population genetics in its central-European range should be following set: Cl-1689, Cl-19, Cl-2947, Cl-11923, Cl-226, Cl-171, Cl-13643, Cl-14610, Cl-14592, Cl-14328, Cl-2274, Cl-14228, Cl-11170, Cl-15116.

The newly developed and characterized microsatellite loci for *C. leucogrammus* will be used in studies on the population structure of this weevil and may provide valuable information for the conservation of its populations.
